# Comparison of Treatment Goals between Users of Biological and Non-Biological Therapies for Treatment of Psoriasis in Japan

**DOI:** 10.3390/jcm10245732

**Published:** 2021-12-07

**Authors:** Yukari Okubo, Ann Chuo Tang, Sachie Inoue, Hitoe Torisu-Itakura, Mamitaro Ohtsuki

**Affiliations:** 1 Department of Dermatology, Tokyo Medical University, 6-1-1 Shinjuku, Shinjuku-ku, Tokyo 160-8402, Japan; yukari-o@tokyo-med.ac.jp; 2 Eli Lilly Japan K.K., Akasaka Garden City 13F, 4-15-1 Akasaka, Minato-ku, Tokyo 107-0052, Japan; 3 Crecon Medical Assessment Inc., 2-12-15 Shibuya, Shibuya-ku, Tokyo 150-0002, Japan; inoue@crecon.co.jp; 4 Eli Lilly Japan K.K., Lilly Plaza One Bldg., 5-1-28, Isogamidori, Chuo-ku, Kobe 651-0086, Japan; itakura_hitoe@lilly.com; 5 Department of Dermatology, Jichi Medical University, 3311-1 Yakushiji, Shimotsuke 329-0498, Tochigi-ken, Japan; mamitaro@jichi.ac.jp

**Keywords:** cross-sectional studies, health care surveys, Japan, psoriasis, biologics, treatment goal

## Abstract

Background: Previously, our cross-sectional observational study in Japan revealed high (68%) discordance within treatment goals between psoriasis patients and their physicians. Objective: This secondary analysis aimed to determine whether patient and physician users of biologics have higher treatment goals than users of non-biologics. Methods: A survey for both patients and physicians on background characteristics, disease severity, treatment goals, treatment satisfaction, and health-related quality of life was conducted at 54 sites. Association between treatment goals and biologic/non-biologic users was assessed using ordinal logistic regression models. Results: In total, 449 patient-physician pairs agreed to participate; 425 completed the survey and were analyzed. More biologic users than non-biologic users reported complete clearance (Psoriasis Area and Severity Index 100) as a treatment goal (patient-reported: 23.6% vs. 16.1%; physician-reported: 26.9% vs. 2.2%). Biologic users were significantly associated with higher treatment goals than non-biologic users (patient-reported: 1.8 (1.15–2.87) (odds ratio (9 5% CI)), *p* = 0.01; physician-reported: 11.0 (5.72–21.01), *p* < 0.01). Among biologic users, higher treatment goals were associated with higher treatment satisfaction (patient- and physician-rated); lower treatment goals were associated with back lesions and increasing patient age (patient-rated) and higher disease severity (physician-rated). Conclusion: Use of biologics among patients with psoriasis was associated with higher treatment goals. Further use of biologics contributed to treatment satisfaction. Appropriate treatment goals that are shared among patients and their physicians may improve treatment outcomes.

## 1. Introduction

Psoriasis is an immune-mediated skin condition that commonly manifests as inflamed, scaly skin lesions [1,2]. Severe symptoms associated with psoriasis are a major contributor to patients’ health-related quality of life (HRQOL) [3,4,5,6]. Biological therapies have emerged as an effective class of treatment for patients with psoriasis that have a significant effect on disease severity [7,8] and are associated with higher levels of treatment satisfaction compared with other therapies [7]. In Japan, the use of biologics is recommended for patients with poor Health Related Quality of Life, HRQOL (Dermatology Life Quality Index (DLQI) score ≥10) [9].

Establishment of treatment goals for patients with psoriasis is considered critical for setting treatment expectations and improving management practices [10]. However, the treatment goals currently recommended in treatment guidelines for psoriasis focus on clinical measures of disease severity and do not take into account other factors such as treatment satisfaction and HRQOL [10,11]. Treatment goals that are aligned between patients and their physicians have the potential to improve treatment outcomes, adherence, and satisfaction [12,13]. Despite this, treatment goals for psoriasis appear to vary widely between patients and their physicians [13,14].

Recently, we conducted a nationwide, cross-sectional observational study in Japan to assess the alignment of treatment goals between patients with psoriasis and their physicians [14]. There was a high level (68%) of discordance of treatment goals between the patient-physician pairs. Factors that contributed most to the discordance were high expectations by patients for complete clearance, and physicians’ perceptions that patients had a low understanding of their treatment options. In addition, we found that more patients in the misaligned group than in the aligned group had not received a prescription for a biologic within the past 2 to 3 weeks (78.3% vs. 66.2%, *p* = 0.008), suggesting that patient and physician biologic users are more aligned in their treatment goals.

For this secondary analysis, we hypothesized that patient and physician users of biologics have higher treatment goals than non-biologic users. To test this hypothesis, we examined the associations between treatment goals among paired patients and physicians who used biological therapies to treat psoriasis versus those who did not.

## 2. Materials and Methods

### 2.1. Study Design

This nationwide, multicenter, cross-sectional observational study was conducted between October 2015 and May 2016 at 54 sites in Japan [14]. The sites included general practitioners, clinics, university hospitals, and private and public hospitals. The study protocol was reviewed and approved by the Ethics Committee of Jichi Medical University, the Central Institutional Review Board of Medical Corporation Ganka-Koseikai, and the relevant local institution ethics committees of each participating hospital. The study protocol was implemented in accordance with the Declaration of Helsinki (2013), the Guidelines for Good Pharmacoepidemiology Practices (2015), the Ethical Guidelines Concerning Medical Studies in Human Subjects in Japan [15], and ethical principles based on the relevant statutes/standards in Japan.

All treatment decisions and clinical assessments were made at the discretion of the treating physicians. Patients who participated in the study gave written informed consent for the collection and use of their information to be included in this study. Informed consent was obtained from patients after physicians had explained the study protocol to the recruited patients. Only patients who gave their informed consent were given the surveys.

### 2.2. Study Population

Study participants were patients with physician-reported moderate-to-severe psoriasis and a history of systemic treatments, including biological drugs, and their treating physicians. Dermatologists with experience in oral or biological treatments for psoriasis patients were included. Patients were not included in the study if they were participating in a clinical trial, had completed a clinical trial less than 6 months before the current study, or had pustular psoriasis, erythrodermic psoriasis, or psoriatic arthritis.

### 2.3. Study Survey

As previously reported [14], the survey comprised 52 questions for patients and 31 questions for physicians. The questions were categorized into background characteristics of patients and physicians, disease severity (Patient Global Assessment (PtGA), Physician Global Assessment (PGA)), treatment goals (same question for both patients and physicians), and treatment satisfaction and HRQOL. Measures used for treatment satisfaction and HRQOL included the Treatment Satisfaction Questionnaire for Medication (TSQM) [16], the Treatment Satisfaction scale (numerical rating scale 0 to 10), the DLQI [17], and the Itch Numeric Rating Scale (Itch NRS) [18]. A DLQI score ≥ 10 was considered as one of the measures for moderate-to-severe psoriasis [19,20]. Treatment goals were categorized from 1 (highest goal) to 7 (lowest goal) where 1 = complete clearance (Psoriasis Area and Severity Index (PASI) 100) [21], 2 = almost complete clearance (PASI 90 to <100), 3 = complete clearance at specific sites (nails, head, genitals, other), 4 = improvement from previous treatment but without complete or almost complete clearance, 5 = relief from itchiness, 6 = other goals, and 7 = no particular goal set.

The ordinal scale for understanding of disease and treatment choice ranged from not at all, to does not understand very well, neither, somewhat understands, understands very well.

Patients who qualified for inclusion were recruited into the study by their physicians. Patients were sent a paper-based survey within 2 weeks of enrollment and returned the surveys by mail. Each patient and their treating physician completed the surveys independently. To minimize the potential for selection bias by physicians, patients were enrolled consecutively.

### 2.4. Statistical Analysis

Variables for each patient-physician pair were grouped into users of biological therapies (biologic users) and those who did not use biological therapies (non-biologic users), and were examined for associations with treatment goals. Biologic users were defined as patients (and their paired physicians) who were currently using a biological drug for treatment of psoriasis or who had used a biological drug within 3 weeks of completing the survey. Analyses comprised the following: Step 1, variables selected from the survey were categorized into biologic user and non-biologic user groups and were evaluated for differences. Categorical variables were evaluated using the chi-square test; continuous variables were evaluated using the Wilcoxon rank-sum test or Student’s *t* test. Step 2, variables selected from the survey were evaluated by ordinal logistic regression to evaluate any correlations with treatment goals. Step 3, variables that were statistically significant (*p* < 0.05) in Steps 1 and Steps 2 were included as covariates. Step 4, of the variables identified in Step 3, only one variable was selected for the same survey question and included in the final covariates for the further analyses. A clinician was asked to review the variables and select one variable that made the most clinical sense. The reasons for setting this rule were: (1) to avoid too many covariates in a stepwise multivariate model; and (2) to avoid including multiple answers from the same question in the multivariate analyses which would be difficult to interpret.

The ordinal logistic regression models assessed the association between treatment goals (outcome variable) and biologic users and non-biologic users (explanatory variables), and were adjusted for covariates. Associations between treatment goals and use of biological therapies were reported as odds ratios (OR) and 95% CIs. Differences between groups were regarded as statistically significant for *p* < 0.05.

All statistical analyses were performed using SAS^®^ Version 9.4 (SAS Institute Inc., Cary, NC, USA).

## 3. Results

### 3.1. Variable Selection

The variables used in the patient-reported analyses included lesion site—symptom on “back” (yes/no), TSQM score (ordinal scale), and DLQI scores (ordinal scale) for daily activities, leisure, and personal relationships. The variables included in the physician-reported analyses included lesion site—symptom on “upper limb” (yes/no), physician’s specialty—psoriasis (yes/no), physician’s workplace (categorical scale), physician’s experience with biologics (ordinal scale), physician’s perspective on the patient’s understanding of their disease (ordinal scale) and treatment choice (ordinal scale), PGA disease severity (0 to 5 scale), and Treatment Satisfaction (0 to 10 scale). Patient age was included in both patient- and physician-reported models because of its clinical importance. Physicians and patients with missing data for treatment goal or who responded that the treatment goal was “other” or “no setting” were excluded from these analyses. Physicians and patients with missing data for the selected covariates were excluded.

### 3.2. Study Population

Of the 449 patient-physician pairs that agreed to participate in the study, 425 (94.7% response rate) completed the survey and were analyzed. Of the included patients, most were male and had a reasonably long disease duration (mean, 18.8 years) (Table 1). For most patients, psoriasis predominantly affected the head, neck, and lower limbs, and most had at least 3% of their body surface area (BSA) affected (Table 1). Most patients were currently being treated with topical medication, and 25.6% of patients were being treated with biologics at the time the survey was conducted. Of the included physicians, most had considerable experience treating patients with psoriasis; 69.6% specialized in psoriasis, 86.8% had 10 or more years’ experience treating patients with psoriasis, and 75.0% saw 20 or more patients per month (Table 2). Treatment Satisfaction and assessment of disease severity (PtGA, PGA) were similar between patients and physicians (Table 1 and Table 2).

#### Results by Biologic vs. Non-Biologic Users

When patients and their paired treatment physicians were compared by biologic versus non-biologic users, we found the following results. There were statistically significant differences between biologic users and non-biologic users for both patient-reported and physician-reported characteristics (Table 3). Biologic users had significantly higher treatment satisfaction based on TSQM (global satisfaction score; 68.6 vs. 57.3, *p* < 0.001) and significantly higher HRQOL scores (lower DLQI scores) than non-biologic users (Table 3). Physicians treating biologic users had significantly greater Treatment Satisfaction (7.8 vs. 6.0, *p* < 0.001) and significantly lower physician-rated disease severity (PGA disease severity 2.0 vs. 2.7, *p* < 0.001) (Table 3). Significantly more physician biologic users were psoriasis specialists, who worked in university hospital settings and had more years’ experience than non-biologic user physicians.

Biologic users contributed to a small proportion (13.8%, 8/58) of the total number of patients with DLQI score ≥ 10 (one of the criteria for “moderate-to-severe” psoriasis). For patients with a DLQI score ≥ 10, non-biologic users had more severe disease (3.18 vs. 2.25, PGA disease severity) and lower treatment satisfaction (4.50 vs. 5.88, patient treatment satisfaction) than biologic users. For physicians of patients with a DLQI score ≥ 10, non-biologic users had less experience with biologics than biologic users (36% vs. 12.5% of physicians with ≤2 years of experience).

### 3.3. Treatment Goals by Biologic vs. Non-Biologic Users

**Figure 1:**
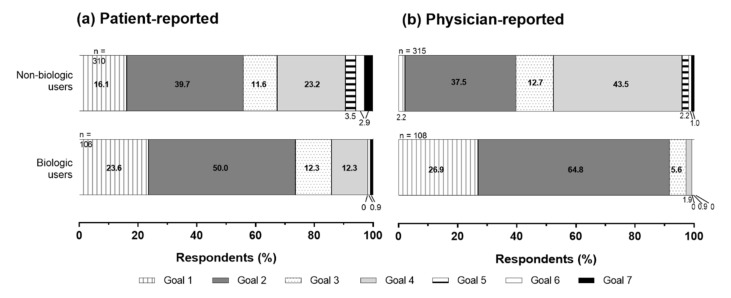
Patient- and physician-reported treatment goals. Goal 1 = complete clearance (Psoriasis Area and Severity Index (PASI) 100) [21], Goal 2 = almost complete clearance (PASI 90 to <100), Goal 3 = complete clearance at specific sites (nails, head, genitals, other), Goal 4 = improvement from previous treatment, but without “complete” or “almost complete clearance”, Goal 5 = relief from itchiness, Goal 6 = other goals, and Goal 7 = no particular goal set. (**a**) A larger number of patient (23.6% vs. 16.1%) and (**b**) physician (26.0% vs. 2.2%) among biologic users had higher treatment goals of achieving complete clearance.

Most patients and physicians reported that their treatment goals were to achieve almost complete clearance, irrespective of whether or not they were biologic users (Figure 1). However, patient and physician biologic users had higher treatment goals than non-biologic users (Figure 1). The percentage of patients reporting complete clearance (PASI 100) as a treatment goal was 23.6% for biologic users and 16.1% for non-biologic users. The percentage of physicians reporting complete clearance (PASI 100) as a treatment goal was 26.9% for biologic users and 2.2% for non-biologic users.

### 3.4. Factors Associated with Treatment Goals by Biologic vs. Non-Biologic Users

Findings from the ordinal analyses showed that patient and physician biologic users were significantly associated with higher treatment goals than their non-biologic user counterparts (Table 4). For patient-reported analyses, biologic users had 1.8-fold higher odds of having higher treatment goals than non-biologic users (OR 1.820 (95% CI 1.154, 2.868), *p* = 0.01). Higher treatment goals were significantly associated with higher patient-rated TSQM scores (global satisfaction) (Table 4). In contrast, lower treatment goals were significantly associated with the presence of back lesions and increasing patient age (Table 4).

For the physician-reported analyses, biologic users had 11.0-fold higher odds of having higher treatment goals than non-biologic users (OR 10.967 (95% CI 5.723, 21.014), *p* < 0.001). Higher treatment goals among biologic users were significantly associated with higher physician-rated Treatment Satisfaction, whereas lower treatment goals were associated with higher PGA disease severity (Table 4).

## 4. Discussion

In our previous primary analysis, which focused on treatment goal alignment and showed that there is a high level of discordance between Japanese patients with psoriasis and their physicians, we showed that patients tended to set higher treatment goals than their physicians and had a greater desire for “complete clearance”, irrespective of treatment received [14]. We also found that there was more treatment goal misalignment (*n* = 220, 78.3%) than alignment (*n* = 88, 66.2%) among non-biological users (i.e., patients who had not had a prescription for a biological drug within the previous 2 to 3 weeks) (*p* = 0.008) [14]. Hence, we extend these findings to show the characteristics of biologic users and non-biologic users and to examine the treatment goals for both patient and physician biologic users versus non-biologic users, adjusted for other factors. In this subgroup analysis of biologic users versus non-biologic users, complete clearance (PASI 100) was reported as a treatment goal for 23.6% and 26.9% of patient and physician biologic users, respectively, compared with 16.1% and 2.2% of patient and physician non-biologic users, respectively. Both patients and physicians who were biologic users set higher treatment goals than non-biologic users. The results of these secondary analyses may explain the difference in treatment goal alignment and misalignment among recent non-biologic users found in the previous primary analysis. Since the introduction of biologics for treating psoriasis, clinicians’ expectations for a successful treatment outcome with biological therapies have increased to “complete” or “almost complete clearance” (PASI > 90) [13]. In addition, patients treated with biologics have greater success achieving their treatment goals [22] and have higher treatment satisfaction compared with other therapies [7,23]. Therefore, given the experience of patients and physicians with biologics, it is likely that both patients and their physicians have higher expectations for treatment success with biologics than with non-biologics.

In our study, there was a larger percentage of male than female patients with psoriasis. In Japan, the higher percentage of males is consistent with the real-world report [1,2,24]. As for how our sample has an even higher percentage of males, it is not known. Although physicians assess the severity of psoriasis based on symptoms and the body area affected, patients can be more focused on the effects of psoriasis on their HRQOL [10,11,25]. Our findings are consistent with this focus on HRQOL in that treatment goals were associated with disease severity and treatment satisfaction for both patient and physician biologic users. At the time this survey was conducted, both patient- and physician-rated disease severity (PtGA, PGA) was lower and treatment satisfaction was higher among biologic users than non-biologic users. This suggests that as disease severity decreases with the use of biologics, treatment satisfaction increases and the clinical improvement experienced by patients translates to greater patient HRQOL.

In the current study, we found that patient biologic users contributed a small proportion (13.8%) of the total number of patients with DLQI scores ≥10, a criterion to be recommended for biological therapies in Japan [9]. Many patients who used non-biologics in our study had high DLQI scores ≥10 and tended to be less satisfied with their treatment than biologic users. In addition, their physicians tended to have less experience with biologics. Together, these data suggest a large number of patients with psoriasis may be undertreated in Japan, despite being eligible for biological therapy.

In the additional analysis in this study focusing on treatment satisfaction misalignment between patient and physician, we reported that “not changing the treatment goal from start of treatment” was a factor in a patient’s treatment satisfaction being higher than that evaluated by the physician [26]. Hence, the importance of the treatment goal leading to satisfactory treatment outcomes would need to be discussed and emphasized.

As this was a nationwide, multicenter, cross-sectional study conducted in real-world clinical practice, the findings are representative of general Japanese patients and their treating physicians. However, there are a few limitations worthy of mention. First, the interpretation of the findings should take into account the cross-sectional design of the study, which limits the strength of any observed associations, the potential for selection bias arising from consecutive enrollment of patients. In a cross-sectional setting, we cannot make an inference on the causal-effect relationships between outcomes of disease state versus setting a high goal and biologics usage. The patient disease state at the time of the survey could have been a result of prior medical interventions. Second, along with setting PASI scores as the treatment goal target, PASI scores would need further validation as a clinically meaningful treatment goal in the future. Although both physicians and patients may consider biologics to have a higher chance of achieving complete or almost complete response, this perspective could be more dominant among physicians. Patients may simply consider the results satisfactory, even being far from complete clearance. Third, as this was a secondary analysis that focused on a comparison between biologic users and non-biologic users, the sample size for some comparisons may not have been sufficiently powered to detect differences between groups. Fourth, since our sample is derived from the Japanese population within the Japanese health care setting, the study should take into consideration that biologics prescribers in Japan are limited to only hospitals or clinics accredited by the Medical Society. Therefore, although patients and their physicians may have higher treatment goals, some prescribers are limited in the use of biologics by this unique Japanese guideline unless there is a solid referral system. In this study, our analysis was to compare “biologic users versus non-biologic users”, and not “biologic accredited versus non-accredited institutions”. We recognized this Japanese health care situation. Therefore, without including the analyses in the main results, we also looked at how the treatment goals performed in the accredited medical institutions versus those in the non-accredited medical institutions. We found that, similar to results in our previous report (5) and in this report, patients and doctors who were biologics users had higher treatment goals (almost complete or complete clearance) than the non-biologic users even when compared only within the accredited institutions (patients: 73% vs. 52%; doctors: 91.4% vs. 41%). In addition, the treatment goal misalignment was higher among pairs of patients and their treating doctors who were not treated with biologics (75%) vs. those treated with biologics (57%) within the accredited institutions. The rate of misalignment among non-biologic users in the accredited institutions (75%) was comparable to the non-biologic users in the non-accredited institutions (69%). Although we found similar trends of treatment goals when comparing the results by categories of biologic accredited institutions, we did not perform in-depth analyses to control the intended associations with other factors due to sample size limitations. Further studies would be needed to further address the differences of treatment goals by health care settings. Finally, while the patient-centric treatment approach is ideal in clinical settings, we should note that treatment satisfaction related to the use of biologics has financial implications to the patients and the health care system or vice versa. This can be a limitation in some health care systems.

In summary, we found that there were differences between biologic users and non-biologic users such as disease severity, physicians’ experience and workplace, and physicians’ perspectives on their patients’ understanding of disease and treatment options. Patients with psoriasis and their physicians who were users of biological therapies shared higher treatment goals after adjustment for contributing factors. Better understanding of treatment goals between patients and their physicians has the potential to contribute to the development of patient-centered goals that can improve treatment outcomes. The results indicate that availability and experience with biologic treatment are elevating treatment goals for both physicians and patients and are addressing unmet treatment needs.

## Figures and Tables

**Table 1:** Patient characteristics.

Variable	Value (*n* = 414) ^5^
Male, %	74.9%
Age (range), y	56.2 ± 13.9 (20.0–93.0)
BMI (range), kg/m^2^	24.3 ± 4.6 (16.0–54.9)
Age at disease onset (range), y	37.2 ± 16.2 (0.0–81.0)
Age at disease diagnosis (range), y	40.0 ± 16.2 (4.0–81.0)
Disease duration from onset (range), y	18.8 ± 11.7 (0.0–65.0)
Body part affected (top 3 nominated)	
Lower limbs	78.0%
Head	70.8%
Back	67.1%
Body surface area affected ^1^	
<1%	24.4%
1–2%	22.0%
3–10%	37.0%
>10%	16.5%
Current treatment received ^2^	
Topical	82.4%
Oral	53.6%
Ultraviolet light	19.1%
Biologic	25.6%
Other	1.4%
Treatment Satisfaction ^3^	6.75 ± 2.27
PtGA disease severity ^4^	2.54 ± 1.26

^1^ Palm size is equivalent to 1%; ^2^ multiple answers were allowed; ^3^ 0 = lowest treatment satisfaction, 10 = highest treatment satisfaction; ^4^ 0 = lowest, 5 = highest severity, all values are mean ± standard deviation unless otherwise indicated. BMI, body mass index; PtGA, Patient Global Assessment; y, year. ^5^ Out of the total sample (*n* = 425), 9 pairs were excluded where patient treatment goal information was missing and 2 pairs were excluded where physician treatment goal information was missing.

**Table 2:** Physician characteristics.

Variable	Value ^5^ (*n* = 70)
Male, %	64.3%
Age (range), y	50.6 ± 11.7 (30.0–80.0)
Specialty ^1^ Psoriasis Allergy Other	69.6% 40.6% 41.8%
Treatment experience with psoriasis ^2^ <2 y 2 ≤ 4 y 4 ≤ 6 y 6 ≤ 8 y 8 ≤ 10 y ≥10 y	0.0% 2.9% 5.9% 4.4% 0.0% 86.8%
Number of patients seen per month ^2^ <5 5–9 10–14 15–19 ≥20	1.5% 5.9% 10.3% 7.4% 75.0%
Treatment Satisfaction ^3^	6.46 ± 2.08
PGA disease severity ^4^	2.51 ± 1.15

^1^ Multiple answers were allowed; ^2^ physician responses with obvious errors and inconsistencies were excluded from the analyses; ^3^ 0 = lowest treatment satisfaction, 10 = highest treatment satisfaction; ^4^ 0 = lowest severity, 5 = highest severity. ^5^ The total number of physicians (*n* = 70) paired to 425 patients. All values are mean ± standard deviation (range) unless otherwise indicated. PGA, Physician Global Assessment; y, year.

**Table 3:** Comparison of patient- and physician-reported characteristics between biologic users and non-biologic users.

Characteristic ^1^	Biologic Users	Non-Biologic Users	*p* ^2^
Patient-reported	*n* ^1^ = 104	*n* ^1^ = 292	
Patient age, y	56.3 ± 15.1	55.9 ± 13.4	0.807
Lesion site, back, *n* (%)	54 (51.9)	211 (72.3)	<0.001
TSQM score (global satisfaction)	68.6 ± 19.6	57.3 ± 17.1	<0.001
DLQI			
DLQI total score	3.2 ± 5.0	5.0 ± 5.3	<0.001
Daily activities	0.5 ± 1.3	1.0 ± 1.5	<0.001
Leisure	0.5 ± 1.1	0.7 ± 1.3	0.044
Personal relationships	0.2 ± 0.9	0.4 ± 1.1	0.028
Physician-reported	*n* ^2^ = 107	*n* ^2^ = 309	
Patient age, y	56.9 ± 15.2	56.4 ± 13.8	0.709
Location of lesion (upper limb), *n* (%)	42 (39.3)	229 (74.1)	<0.001
Physician’s specialty—psoriasis, *n* (%)	99 (92.5)	231 (75)	<0.001
Physician’s workplace, *n* (%)			<0.001
Clinic	35 (32.7)	202 (65.4)	
University hospital	47 (43.9)	81 (26.2)	
Other	25 (23.4)	26 (8.4)	
Physician’s experience—biologics, *n* (%)			<0.001
None	0 (0.0)	77 (24.9)	
<1 y	0 (0.0)	10 (3.2)	
1 ≤ 2 y	9 (8.4)	25 (8.1)	
2 ≤ 3 y	4 (3.7)	14 (4.5)	
3 ≤ 4 y	13 (12.2)	19 (6.2)	
4 ≤ 5 y	35 (32.7)	58 (18.8)	
>5 y	46 (43)	106 (34.3)	
Physician’s perspective on patient’s understanding of disease, *n* (%)			0.001
Understands very well	42 (39.3)	64 (20.7)	
Somewhat understands	55 (51.4)	215 (69.6)	
Neither	7 (6.5)	27 (8.7)	
Does not understand very well	3 (2.8)	3 (1.0)	
Does not understand at all	0 (0.0)	0 (0.0)	
Physician’s perspective on patient’s understanding of treatment choice, *n* (%)			<0.001
Understands very well	45 (42.1)	69 (22.3)	
Somewhat understands	49 (45.8)	209 (67.6)	
Neither	10 (9.4)	27 (8.7)	
Does not understand very well	3 (2.8)	4 (1.3)	
Does not understand at all	0 (0.0)	0 (0.0)	
PGA disease severity	2.0 ± 1.5	2.7 ± 1.0	<0.001
Treatment Satisfaction (0–10 scale)	7.8 ± 1.7	6.0 ± 2.0	<0.001

^1^ From the total 425, the number of biologic and non-biologic user pairs that remained in patient-reported analyses was *n* = 396. (The following were excluded: 9 pairs with missing patient treatment goal information, 20 pairs with patients who reported treatment goal as “other” or “no setting”). ^2^ From the total 425, the number of biologic and non-biologic user pairs in physician-reported analyses was *n* = 416. (The following were excluded: 2 pairs with missing information on treatment goal and 7 pairs who reported that the treatment goal was “other” or “no setting”). Categorical variables were compared using the chi-square test and continuous variables were compared using the *t* test or Wilcoxon rank-sum test. Data are mean (standard deviation) unless otherwise indicated. DLQI, Dermatology Life Quality Index; PGA, Physician Global Assessment; TSQM, Treatment Satisfaction Questionnaire for Medication; y, years.

**Table 4:** Regression analysis of factors associated with treatment goals.

Variables ^1,2^	Odds Ratio (95% CI)	*p*
Patient-reported variables v ^3^	*n* = 383	
Biologic vs. non-biologic	1.820 (1.154, 2.868)	0.010
Age, y	0.983 (0.970, 0.997)	0.016
Lesion site, back	0.544 (0.358, 0.825)	0.004
TSQM score (global satisfaction)	1.014 (1.002, 1.026)	0.021
DLQI score		
Daily activities	1.106 (0.900, 1.360)	0.336
Leisure	1.132 (0.886, 1.446)	0.321
Personal relationships	1.085 (0.858, 1.373)	0.497
Physician-reported variables ^4^	*n* = 413	
Biologic vs. non-biologic user	10.967 (5.723, 21.014)	<0.001
Patient age, y	1.003 (0.989, 1.017)	0.664
Location of lesion (upper limb)	0.870 (0.549, 1.377)	0.552
Physician’s specialty—psoriasis	1.042 (0.602, 1.803)	0.884
Physician’s workplace		
University hospital	0.696 (0.429, 1.128)	0.141
Other	0.663 (0.342, 1.284)	0.223
Physician’s experience—biologics	1.096 (0.991, 1.212)	0.073
Patient’s understanding of disease (physician perspective)	1.390 (0.839, 2.303)	0.202
Patient’s understanding of treatment choice (physician perspective)	0.631 (0.386, 1.032)	0.067
PGA disease severity	0.720 (0.584, 0.887)	0.002
Treatment Satisfaction	1.285 (1.139, 1.449)	<0.001

^1^ From the *n* = 396 of Table 3, 383 remained in the analyses (13 pairs that had missing covariate information were excluded). ^2^ From the *n* = 416 of Table 3, 413 remained in the analyses (3 pairs that had missing covariate information were excluded). ^3^ *p* values for differences in treatment goals between biologic and non-biologic users were calculated using an ordinal logistic regression model adjusted for age (y), lesion site—back (yes/no), TSQM score (ordinal scale), and DLQI scores (ordinal scale). ^4^ *p* values for differences in treatment goals between biologic and non-biologic users were calculated using an ordinal logistic regression model adjusted for age (y), lesion site—upper limb (yes/no), Physician’s specialty—psoriasis (yes/no), Physician’s workplace (yes/no), Physician’s experience with biologics (ordinal scale), Physician’s perspective of patient’s understanding of disease (ordinal scale) and treatment choice (ordinal scale), Physician GA severity (0–5 scale), and Treatment Satisfaction (0–10 scale). DLQI, Dermatology Life Quality Index; PGA, Physician Global Assessment; TSQM, Treatment Satisfaction Questionnaire for Medication; y, years.

## Appendix A. List of Participating Institutions

Department of Dermatology, Asahikawa Medical University
Department of Dermatology, Jichi Medical University
Department of Dermatology, Tokyo Women’s Medical University
Department of Dermatology, School of Medicine, Teikyo University
Department of Dermatology, Tokyo Medical University
Department of Dermatology, School of Medicine, Tokai University
Department of Dermatology, Graduate School of Medicine, Gifu University
Graduate School of Medical Sciences, Nagoya City University
Department of Dermatology, School of Medicine, Kindai University
Department of Dermatology, Graduate School of Medicine, Osaka City University
Department of Dermatology, Kawasaki Medical School
Department of Dermatology, Faculty of Medicine, Fukuoka University
Department of Dermatology, Tokyo Teishin Hospital
Department of Dermatology, St Luke’s International Hospital
Department of Dermatology, Yokohama Chuo Hospital
Public Interest Incorporated Foundation Jiai-kai, Branch of Imamura Hospital
Department of Dermatology, Ina Central Hospital
Department of Dermatology, Iida Municipal Hospital
Department of Dermatology, Osaka Kaisei Hospital
Medical Corporation Kojin-kai, Sapporo Dermatology Clinic
Medical Corporation Kojin-kai, Fukuzumi Dermatology Clinic
Kobayashi Skin Clinic
Department of Dermatology, EST Clinic
Sugawara Dermatology Clinic
Medical Corporation Subaru-kai, Sugai Dermatology Park Side Clinic
Hattori Dermatology Clinic
Medical Corporation Koten-kai, Iidabashi Clinic
Medical Corporation Shohei-kai, Niki Dermatology Clinic
Clinic of Dermatology, Ningyocho
Dr. Mariko Skin & Dermatology Clinic
Tsujimoto Skincare Clinic
Shirosaki Dermatology & Neurology Clinic
Kato Dermatology
Hou Dermatology
Machino Skin Clinique
Yasumoto Dermatology Clinic
Takagi Dermatology Clinic
Fushimi Skin Clinic
Omorimachi Dermatology
Hayashibe Derma Clinic
Hasegawa Dermatology Clinic
Medical Corporation Kojin-kai, Ario Sapporo Dermatology Clinic
Atago Dermatology
Medical Corporation Shotoku-kai, Hino Clinic
Nomura Dermatology Clinic
Zoshiki Dermatology Clinic
Nakatsu Dermatology Clinic
Saruwatari Dermatology Clinic
Kusuhara Dermatology Clinic
Medical Corporation Shimizu Dermatology Clinic
Kokubu Clinic, Abashiri Dermatology Clinic
Nishide Skin Clinic
Kazama Skin Clinic
Shimizu Skin Clinic
